# LoopID reveals condensate-mediated enhancer–promoter interaction regulates cell fate

**DOI:** 10.1093/lifemedi/lnag019

**Published:** 2026-06-30

**Authors:** Shaoshuai Jiang, Lu Liu, Xinyi Liu, Junjun Ding

**Affiliations:** Center for Stem Cell Biology and Tissue Engineering, Key Laboratory for Stem Cells and Tissue Engineering, Ministry of Education, Department of Histology and Embryology, Zhongshan School of Medicine, Sun Yat-sen University, Guangzhou 510080, China; Center for Stem Cell Biology and Tissue Engineering, Key Laboratory for Stem Cells and Tissue Engineering, Ministry of Education, Department of Histology and Embryology, Zhongshan School of Medicine, Sun Yat-sen University, Guangzhou 510080, China; Center for Stem Cell Biology and Tissue Engineering, Key Laboratory for Stem Cells and Tissue Engineering, Ministry of Education, Department of Histology and Embryology, Zhongshan School of Medicine, Sun Yat-sen University, Guangzhou 510080, China; Center for Stem Cell Biology and Tissue Engineering, Key Laboratory for Stem Cells and Tissue Engineering, Ministry of Education, Department of Histology and Embryology, Zhongshan School of Medicine, Sun Yat-sen University, Guangzhou 510080, China

Enhancers are genomic powerhouses that orchestrate precise spatiotemporal gene expression by physically contacting target promoters to form enhancer–promoter (E–P) interactions. These interactions are crucial for defining cellular identity, yet the molecular machinery that establishes and maintains them has remained poorly characterized. In a recent study, we developed a chromatin structure-based proteomic approach—LoopID [1]—to systematically identify the protein constituents of specific E–P interactions, unveiling a previously undefined set of protein assemblies at chromatin loops that we term the looposome. The looposome is highly enriched with epigenetic regulators; histone demethylase JMJD2 (Jumonji domain-containing protein 2, also known as KDM4) governs E–P interactions through biomolecular condensate formation, functioning independently of its catalytic activity. Furthermore, targeted assembly of JMJD2 condensates at defined genomic loci enables *de novo* formation of E–P interactions, driving cell fate transition. Together, these findings represent both a technological and conceptual advance—establishing LoopID as a foundational tool for dissecting chromatin architecture, redefining epigenetic regulators as structural organizers, and offering a new strategy to control cell fate through 3D genome reconfiguration.

## LoopID: the first proteomics approach based on chromatin loop structure

Enhancer–promoter (E–P) interactions, organized by diverse chromatin-associated proteins, are central to gene regulation and cell fate determination. However, identifying the molecular components that occupy these loops has long been technically challenging.

Existing methods for profiling E–P complexes all have notable limitations:

- Identify the candidates from chromatin immunoprecipitation with mass spectrometry (ChIP–MS), using antibodies directed toward histones with modifications characteristic of enhancer and promoter chromatin (H3K27ac and H3K4me3, respectively). However, H3K27ac is also highly enriched on many promoters, and a large number of H3K27ac-labeled enhancers do not interact with promoters. Therefore, the factors that bind non-interactive enhancers or promoters would be identified.
- *In situ* capture of the interactome of an interesting enhancer or promoter by dCas9-mediated affinity purification [2]. However, on one hand, a single enhancer is able to interact with different promoters in individual cells. The interactions are both rare and dynamic, with a fraction of about 3%–6.05% and a median loop lifetime of about 10–30 min. Therefore, these non-structure-based methods prefer to purify the interactome of enhancers or promoters that are not interacting.
- Combine 3C-based technology, such as Hi-C [3], with protein ChIP-seq; the candidates on the E–P interactions can be analyzed through binding motifs. But abundant factors without ChIP-seq data may be lost. To experimentally identify the interactome on the E–P interactions that are really interacting, a structure-based method needs to be developed, which is vital for illustrating the underlying regulatory mechanisms of E–P interactions.

**Figure 1.**
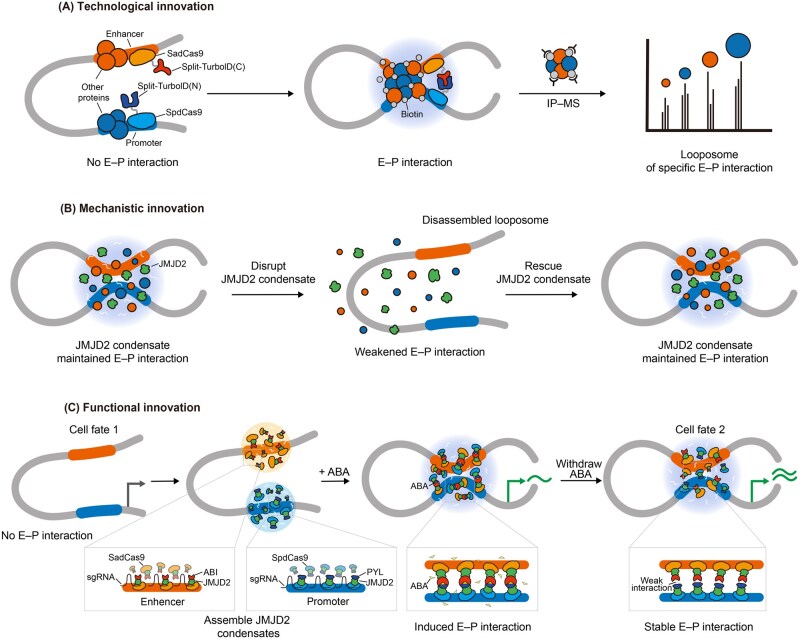
Advances in chromatin loop proteomics, mechanistic insights, and functional applications. (A) Technological advancement—LoopID development. Diagram illustrating LoopID, an integrated platform combining CRISPR–Cas9 with split proximity labeling to map proteins localized at interacting chromatin loop anchors, such as E–P interactions. (B) Mechanistic discovery—catalysis-independent role of epigenetic regulator in modulating chromatin organization. Unveiling that JMJD2 facilitates E–P loop formation through forming biomolecular condensates, independent of its enzymatic demethylase activity, thereby revealing a previously unrecognized structural function of chromatin modifiers. (C) Functional innovation—synthetic control of E–P loops for cell fate engineering. Conceptual illustration of artificially establishing E–P loops via constructing condensates at specific genomic loci, allowing precise modulation of gene expression and cell fate reprogramming.

To overcome these challenges, we developed LoopID [1], a structure-guided proteomic system that integrates CRISPR-Cas9 targeting with a split proximity labeling enzyme (Fig. 1A). In this design, catalytically inactive dCas9 proteins are fused to the N- and C-terminal fragments of a split biotin ligase (Split-TurboID) [4]. Using *Staphylococcus aureus* dCas9 (SadCas9) and *Streptococcus pyogenes* dCas9 (SpdCas9), each directed by distinct guide RNAs to target an enhancer and its corresponding promoter, respectively, LoopID labels proteins only when the two loci physically interact. When an endogenous E–P interaction forms, the two Split-TurboID fragments come into proximity and reconstitute an active enzyme that biotinylates proteins within a few nanometers of the loop anchors. After cell lysis, biotinylated proteins are purified by streptavidin affinity and identified through mass spectrometry.

By focusing on chromatin loop topology rather than linear genomic regions, LoopID enables the selective identification of proteins precisely localized at interacting E–P anchors. This provides the first comprehensive proteomic map of E–P interactions, offering an unprecedented window into the molecular organization of chromatin architecture. Applying LoopID to mouse embryonic stem cells (ESCs), we generated a detailed catalog of looposome proteins, which includes canonical structural regulators (e.g. CTCF and cohesin), transcriptional coactivators, and a striking abundance of epigenetic regulators—a category whose contributions to chromatin structure have been largely underappreciated.

Collectively, LoopID emerges as a foundational technology for decoding the molecular logic of E–P organization and 3D genome folding.

### A catalytic-independent role of epigenetic regulators in chromatin architecture

Among the epigenetic regulators most enriched in the looposome, the histone demethylase JMJD2 (Jumonji domain-containing protein 2) stood out as a crucial player. Traditionally, JMJD2 is known for demethylating histone H3 lysine residues (e.g. H3K9me3, H3K36me3), thus regulating transcription via catalytic activity. However, recent studies hint that many epigenetic enzymes may also perform non-catalytic functions in transcriptional regulation. Previous studies have found that the regulation of chromatin structure by epigenetic regulators depends on their catalytic activity. However, recent studies revealed that the functions of many epigenetic regulators extend beyond their catalytic activities in regulating transcription [5]. Therefore, we further explored the potential for JMJD2 condensates to maintain E–P interactions in a catalytic-independent manner.

Interestingly, we found that JMJD2 forms biomolecular condensates that localize specifically to E–P anchors, facilitating connection stability and function. Acute depletion of JMJD2 leads to a rapid and global weakening of E–P interactions without immediate changes to histone methylation or transcription, positioning it as a critical player in maintaining 3D genome architecture, indicating a structural rather than enzymatic role. Crucially, catalytic mutants of JMJD2 were able to rescue E–P interactions and restore looposome integrity, whereas condensate-defective mutants failed to do so. This demonstrates that JMJD2 regulates chromatin architecture independently of its demethylase activity and that condensate formation is crucial for maintenance of E–P interactions (Fig. 1B).

This finding provides the first direct demonstration that an epigenetic modifier can act as a structural organizer of chromatin loops, rather than solely as a chemical modifier of histones. It redefines the conceptual boundary between chromatin enzymology and 3D genome organization.

### Engineering E–P interaction through constructing biomolecular condensates at specific genomic loci

A particularly exciting implication of this work is the ability to directly manipulate cell fate by engineering E–P interactions through constructing biomolecular condensates at specific genomic loci. By recruiting JMJD2 to defined enhancer or promoter regions, we induced the *de novo* formation of E–P interactions, effectively reprogramming cell identity. In differentiated cells, targeted JMJD2 condensates reinforced pluripotency-specific E–P interactions, facilitating reprogramming into induced pluripotent stem cells. In pluripotent ESCs, the same approach reactivated 2-cell-specific regulatory networks, driving conversion into 2-cell-like cells (2CLCs)—a state associated with totipotent potential (Fig. 1C).

This finding introduces a new dimension to cellular reprogramming: rather than modulating transcription factors or signaling pathways, chromatin architecture itself can be remodeled via condensate assembly to achieve fate transitions. The capacity to construct functional E–P loops through localized phase separation offers a powerful new avenue for regenerative medicine, with potential applications in developmental biology, tissue repair, and modeling chromatin-associated disorders.

### Implications and future perspectives

Our findings open three major frontiers in epigenetics and 3D genomics:

- **Technological innovation**—LoopID represents the only available proteomic approach designed specifically around chromatin loop structures. LoopID is particularly powerful for uncovering chromatin structural regulators within specific cell types or biological contexts. As increasing evidence implicates aberrant chromatin organization in developmental abnormalities and human diseases, LoopID offers a unique approach to map the chromatin structure–associated proteins involved in pathogenic reorganizations.
- **Mechanistic innovation**—The identification of a catalytic-independent function of JMJD2 challenges the conventional enzyme-centric view of chromatin regulation. It underscores that protein condensate behavior, rather than catalytic modification, can be the dominant mechanism controlling 3D chromatin topology.
- **Functional innovation**—The demonstration that biomolecular condensates can be constructed to engineer new E–P interactions at defined loci introduces a revolutionary tool for cell fate engineering. This approach could be harnessed to correct epigenetic defects, restore chromatin organization in disease contexts, or guide somatic cell reprogramming with unprecedented precision.

In the long term, the concept of looposome biology will likely expand beyond E–P interactions. Similar condensate-mediated assemblies may organize other genomic processes—such as replication timing, DNA repair, or chromatin compartmentalization—suggesting a universal principle of genome regulation.

## Concluding remarks

In eukaryotic nuclei, the three-dimensional folding of the genome—particularly E–P communication—acts as a molecular “switch” that governs cell identity. Scientists could visualize chromatin interactions through techniques like Hi-C but could not identify the exact protein complexes stabilizing these dynamic interactions. The looposome thus remained a “black box” of chromatin biology. This work breaks this barrier by introducing LoopID, an innovative technology that bridges chromatin structure and molecular composition. LoopID not only unlocks the “black box” of E–P loop proteins but also provides a high signal-to-noise approach for capturing their dynamic interactomes, filling a long-standing methodological gap in 3D genome research.

Furthermore, the discovery that JMJD2 forms condensates to mediate E–P interactions uncovers the first catalysis-independent role of an epigenetic modifier in organizing chromatin architecture. This discovery not only reveals a novel structural regulator of E–P interactions but also introduces a new concept: the catalytic-independent function of epigenetic modifiers in transcriptional regulation can be mediated through modulating chromatin structure, rather than solely through interactions with other transcriptional regulatory factors.

Finally, by assembling JMJD2 condensates at specific genomic loci, we demonstrate that chromatin interactions—and by extension, cell fate—can be engineered. This ability to rewire the 3D genome through phase separation not only establishes causal links between structure and function but also provides a versatile toolkit for synthetic epigenetics and cellular reprogramming.

In sum, this study represents a milestone at the intersection of 3D genome biology, epigenetic regulation, and synthetic chromatin engineering—defining a new era in our capacity to analyze, interpret, and ultimately design chromatin architecture.

## References

[1] Jiang S , Liu X, Zhang Z et al JMJD2 regulates enhancer–promoter interactions via biomolecular condensate formation. Nat Genet 2026;58:593–606. 41402457 10.1038/s41588-025-02415-8

[2] Liu X , Zhang Y, Chen Y et al In situ capture of chromatin interactions by biotinylated dCas9. Cell 2017;170:1028–43.e1019. 28841410 10.1016/j.cell.2017.08.003 PMC6857456

[3] Rao SSP , Huntley MH, Durand NC et al A 3D map of the human genome at kilobase resolution reveals principles of chromatin ­looping. Cell 2014;159:1665–80. 25497547 10.1016/j.cell.2014.11.021 PMC5635824

[4] Cho KF , Branon TC, Rajeev S et al Split-TurboID enables contact-dependent proximity labeling in cells. Proc Natl Acad Sci U S A 2020;117:12143–54. 32424107 10.1073/pnas.1919528117 PMC7275672

[5] Morgan MAJ , Shilatifard A. Epigenetic moonlighting: Catalytic-independent functions of histone modifiers in regulating transcription. Sci Adv 2023;9:eadg6593. 37083523 10.1126/sciadv.adg6593 PMC10121172

